# Forget the phosphorus: A case of hypervitaminosis D-induced symptomatic hypercalcemia 

**DOI:** 10.5414/CNCS110414

**Published:** 2021-02-09

**Authors:** Todd Nguyen, Deanna Joe, Ankur D. Shah

**Affiliations:** 1 The Warren Alpert Medical School of Brown University,; 2 Department of Internal Medicine,; 3 Division of Nephrology, Rhode Island Hospital, and; 4 Division of Nephrology, Providence VA Medical Center, Providence, RI, USA

**Keywords:** hypervitaminosis D, vitamin D toxicity, altered mental status, hypercalcemia, hypophosphatemia

## Abstract

Hypercalcemia is a frequently encountered electrolyte abnormality with a well-described differential diagnosis and classic algorithm for evaluation. The treatment for hypercalcemia is dependent on the underlying etiology. Hypervitaminosis D is an uncommon cause of hypercalcemia, but the use of vitamin D supplementation has expanded and case reports of supplemental vitamin D induced hypercalcemia have become more frequent. We present a case of hypervitaminosis D-induced altered mental status where diagnosis was delayed and additional invasive testing was performed due to an assumption regarding phosphatemia.

## Introduction 

Vitamin D is a steroid hormone crucial for both bone health and calcium homeostasis [1]. It can be obtained through dietary sources in the form of vitamin D2 (ergocalciferol) and vitamin D3 (cholecalciferol), but the majority of vitamin D3 is synthesized when 7-dehydrocholesterol is exposed to ultraviolet B rays at the epidermis [1]. Vitamin D3 supplementation is commonly used for musculoskeletal health, and there has been an increase in use as studies report health benefits including improvement in symptoms of depression and blood pressure, and decreased respiratory infections and mortality [1, 2]. Although hypervitaminosis D is uncommon, multiple studies have demonstrated toxicity from high dose supplementation [3, 4, 5, 6, 7, 8]. Hypervitaminosis D results in hypercalcemia and classically presents with hyperphosphatemia [9]. Hypercalcemia is broadly divided into parathyroid hormone (PTH)-mediated, vitamin D-mediated, and non-humoral etiologies [9]. Classic symptoms of hypercalcemia include altered mental status, constipation, shortened QT interval, muscle weakness, nephrolithiasis, and renal failure [9]. We present a case report of hypervitaminosis D with hypercalcemia and hypophosphatemia. This case underlines the importance of lessening the reliance on phosphatemia in guiding diagnostic testing. 

## Case report 

A 64-year-old man with a history of chronic obstructive pulmonary disease and ethanol abuse use presented to the emergency department with altered mentation. He was obtunded on arrival, unable to provide any additional history. Presenting vitals were notable for blood pressure of 150/93, pulse of 69 beats per minute, temperature of 97.3 °F (36.3 °C), respiratory rate of 18 BPM, and SpO_2_ 95%. Physical exam revealed bilateral upper extremity tremors and obtundation. Pupils were symmetric and reactive to light, and he periodically tracked with his eyes. Neurological exam, including reflexes, was limited due to altered mental status. Cardiopulmonary exam was benign. Chest X-ray was notable for a normal cardiopulmonary silhouette. Electrocardiogram revealed normal sinus rhythm with a QTc interval of 409 ms. Computed tomography of the brain without contrast showed normal gray-white matter differentiation. 

Laboratory evaluation revealed a sodium 143 mEq/L (135 – 145 mEq/L), potassium 3.0 mEq/L (3.6 – 5.1 mEq/L), chloride 105 mEq/L (98 – 110 mEq/L), bicarbonate 27 mEq/L (22 – 32 mEq/L), blood urea nitrogen 14 mg/dL (6 – 24 mg/dL), creatinine 1.11 mg/dL (0.64 – 1.27 mg/dL), undetectable blood ethanol level, blood glucose 95 mg/dL (67 – 99 mg/dL), calcium 12.8 mg/dL (8.5 – 10.5 mg/dL), ionized calcium 6.3 mg/dL (4.2 – 5.2 mg/dL), phosphorus of 2.2 mg/dL (2.4 – 4.8 mg/dL), and albumin 4.0 g/dL (3.5 – 5.0 g/dL). Initial management consisted of volume expansion with 3 L normal saline without improvement in mentation. Calcitonin was given on hospital day 2 and zoledronic acid was given on hospital day 3, but hypercalcemia persisted. Further labwork returned with intact parathyroid hormone (iPTH) 17 pg/mL (18 – 80 pg/mL), parathyroid hormone related peptide (PTHrP) 9 pg/mL (pg/mL 14 – 27), serum protein electrophoresis (SPEP) and immunofixation (IFE) without evidence of monoclonal protein, κ light chains 85.4 mg/L (3.3 – 19.4 mg/L), λ light chains 40.8 mg/L (5.7 – 26.3 mg/L), and β-2 microglobulin 3.75 mg/L (0.97 – 2.64 mg/L). The elevation of κ/λ ratio in the setting of a normal glomerular filtration rate prompted evaluation for a monoclonal gammopathy. Skeletal survey was negative for lytic lesions and bone marrow biopsy was negative for plasma cell dyscrasia. Finally, the following labs resulted: 25-hydroxy vitamin D > 150 ng/mL (30.0 – 100.0 ng/mL), 1,25-hydroxy vitamin D 36 pg/mL (18 – 72 pg/mL). The patient remained agitated and confused for the first 10 days of hospitalization, but mentation improved thereafter with calcium normalizing after 18 days. Upon recovery, he reported misunderstanding medication instructions and had been consuming 4 vitamin D 50,000 IU tablets daily. 

## Discussion 

This case highlights the risks of pathophysiologic assumptions. The findings of hypercalcemia with appropriately suppressed iPTH and low PTHrP resulted in an evaluation for hypervitaminosis D. However, hypophosphatemia directed the evaluation away from hypervitaminosis D, leading to additional evaluation for paraproteinemia. The finding of an abnormal κ-to-λ ratio and elevated β-2 microglobulin, resulted in evaluation for plasma cell dyscrasia with a bone marrow biopsy. The patient had an elevated vitamin D 25OHD and normal calcitriol revealing the ultimate diagnosis to be hypervitaminosis D. 

The presentation of hypercalcemia frequently varies with the level of serum calcium. Individuals with mild hypercalcemia (< 12 mg/dL) are typically asymptomatic [10]. Moderate hypercalcemia (12 – 14 mg/dL) classically presents with impaired concentration, as well as symptoms of anorexia, constipation, polydipsia, and polyuria [10]. Severe hypercalcemia (> 14 mg/dL) can present with nausea, vomiting, arrhythmia, and altered mental status, and in some cases, may result in coma and death [10]. The patient in our report presented with altered mental status due to hypercalcemia. The most common causes of hypercalcemia are primary hyperparathyroidism and malignancy [9, 11]. The most important diagnostic test is the measurement of parathyroid hormone: high PTH indicates primary hyperparathyroidism, while normal or low PTH indicates hypercalcemia of malignancy [9, 11]. 

Hypervitaminosis D classically presents as hypercalcemia with hyperphosphatemia and low PTH [8, 12]. Vitamin D3 is converted to 25 hydroxy-cholecalciferol (25OHD) in the liver, and subsequently to calcitriol (1,25-dihydroxycholecalciferol (1,25OHD)) in the kidney [2]. The vitamin D receptor can be activated by calcitriol or 25 hydroxy-cholecalciferol at supraphysiological levels [8, 12]. Activation of vitamin D receptors is known to increase small intestine absorption of calcium and phosphorus, and also weakly increases skeletal resorption of calcium and phosphorus, as well as kidney reabsorption of calcium [9]. A prospective study of hypervitaminosis D found that some patients receiving multiple intramuscular injections of vitamin D3 developed hypervitaminosis D with hypophosphatemia [6]. Additionally, there is one case of hypervitaminosis D with hypophosphatemia in an infant receiving liquid supplementation of vitamin D3 [13]. 

Serum phosphorus regulation is likely incompletely understood. Phosphate is regulated by PTH, fibroblast growth factor 23 (FGF23), and 1,25OHD [14]. Phosphate is filtered in the glomerulus, and ~ 85% is reabsorbed in the proximal tubule via type 2a and 2c sodium-phosphate cotransporters [14, 15]. PTH and FGF23 decrease type 2a and 2c sodium-phosphate cotransporters at the proximal tubules and brush border resulting in decreased serum phosphate [14, 15]. Although PTH causes osteoclast-mediated release of phosphate and calcium from bone, there is a net hypophosphatemia due to decreased renal reabsorption [10]. At the intestinal brush border, 1,25OHD promotes expression of type 2b sodium-phosphate cotransporters which causes increased transcellular phosphate absorption resulting in increased serum phosphate [14, 15]. Although we understand that phosphorus levels are mediated by PTH, 1,25OHD, and FGF23, the exact nature of this regulation has not been characterized [10, 14]. Furthermore, how the body senses phosphorus has yet to be elucidated [10, 14]. It is important for hospital providers to recognize that hyperphosphatemia is not always seen in hypervitaminosis D, and though rare, hypervitaminosis D should always be considered in the differential for a patient presenting with hypercalcemia with low PTH. 

## Conclusion 

This case highlights the importance of having a broad differential for hypercalcemia. We recommend that hypervitaminosis D be considered in patients with hypercalcemia and low PTH regardless of phosphorus level. 

## Funding 

The authors did not receive support for this work. 

## Conflict of interest 

The authors declare no conflict of interest. 
